# Supplementary material for: Post-COVID-19 Syndrome in Non-Hospitalized Individuals: Healthcare Situation 2 Years after SARS-CoV-2 Infection
Source: Viruses. 2023 Jun 5;15(6):1326. doi: 10.3390/v15061326 (PMC10303962; doi:10.3390/v15061326)
Supplement: Supplementary file 1 [file viruses-15-01326-s001.zip › viruses-2427782-supplementary/viruses-2427782-supplementary-Figure S1.pptx]

## Slide 1
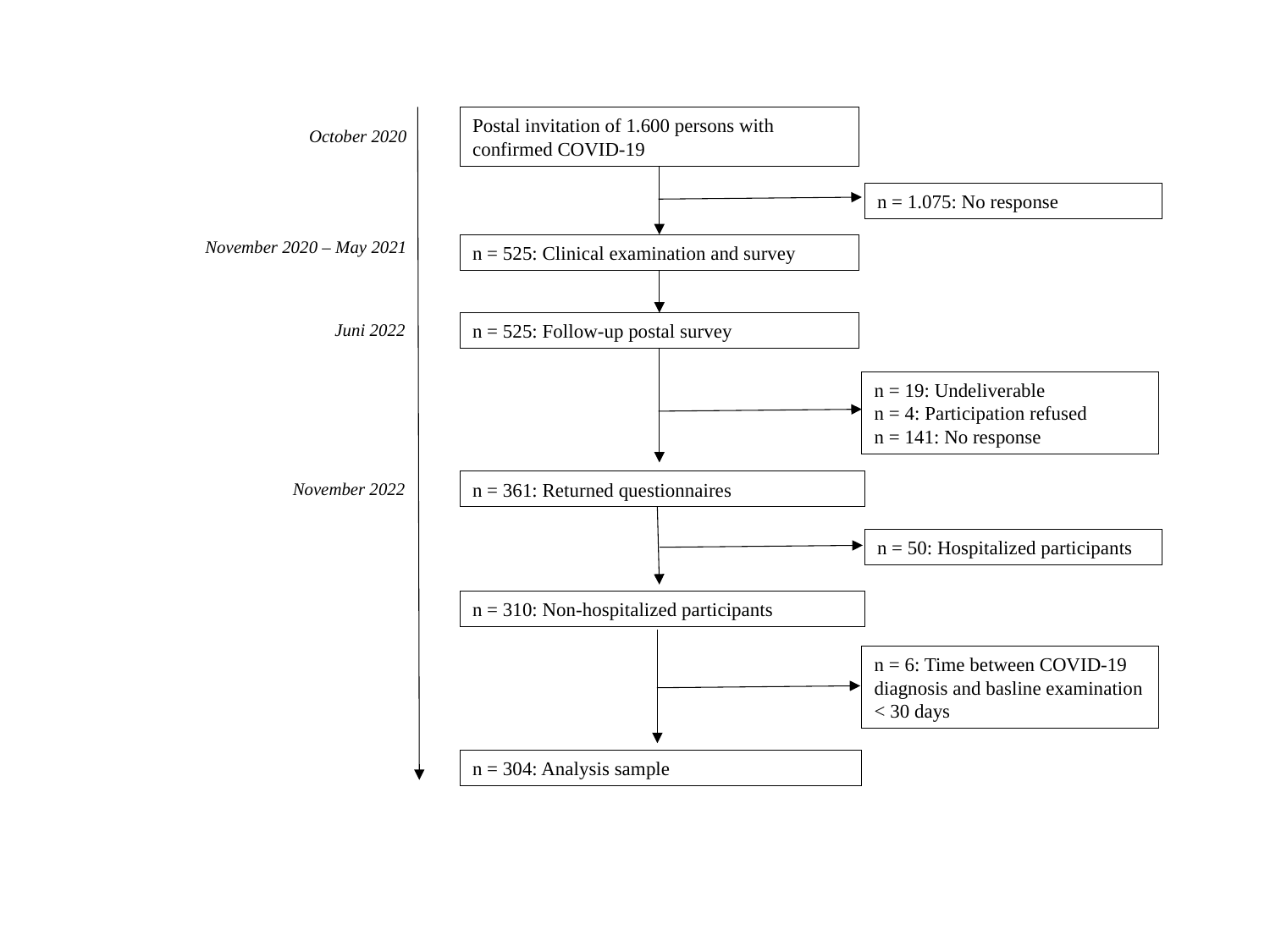

Postal invitation of 1.600 persons with confirmed COVID-19
October 2020
n = 1.075: No response
November 2020 – May 2021
n = 525: Clinical examination and survey
Juni 2022
n = 525: Follow-up postal survey
n = 19: Undeliverable
n = 4: Participation refused
n = 141: No response
November 2022
n = 361: Returned questionnaires
n = 50: Hospitalized participants
n = 310: Non-hospitalized participants
n = 6: Time between COVID-19 diagnosis and basline examination < 30 days
n = 304: Analysis sample
