# Supplementary material for: Post-COVID-19 Syndrome in Non-Hospitalized Individuals: Healthcare Situation 2 Years after SARS-CoV-2 Infection
Source: Viruses. 2023 Jun 5;15(6):1326. doi: 10.3390/v15061326 (PMC10303962; doi:10.3390/v15061326)
Supplement: Supplementary file 1 [file viruses-15-01326-s001.zip › viruses-2427782-supplementary/viruses-2427782-supplementary-Table S1.pdf]

Supplementary Table S1: Frequency of symptoms experienced at baseline examination (median 9 months post diagnosis) and follow-up survey (median 26 months post diagnosis).

	**Baseline (n = 304)**		**Follow-Up (n = 304)**	
	**n**	**%**	**n**	**%**
Fatigue or exhaustion	103	33.89	158	52.8
Concentration problems	82	27.2	105	34.9
Sleep problems	80	26.4	91	30.4
Headache	76	25.1	114	37.6
Dyspnea on exertion	74	24.4	82	27.4
Memory problems	71	23.4	101	33.6
Muscle – or joint pain	67	22.1	127	42.1
Mood swings	66	21.8	80	26.6
Impairment of smell function	62	20.5	50	16.5
Sleepiness	58	19.1	93	31.0
Flatulence	56	18.5	94	31.2
Depressive mood	51	16.8	61	20.3
Impairment of taste function	50	16.5	35	11.6
Heartburn	48	15.8	45	15.1
Rhinitis or running nose	46	15.2	83	27.4
Palptations	45	14.9	55	18.5
Stuffy nose	42	13.9	80	26.4
Cough	34	11.2	69	23.1
Throat pain	32	10.6	36	12.1
Hair loss	30	9.9	32	10.6
Teary eyes	30	9.9	50	16.6
Vertigo	27	8.9	47	15.6
Anxiety, panic	26	8.6	36	11.8
Muscle weakness	25	8.3	45	15.0
Muscle stiffness	24	8.0	50	16.6

Dyspnea on rest-	24	7.9	25	8.4
Diarrhea	22	7.3	46	15.2
Skin rash	21	6.9	22	7.3
Stomach pain	19	6.3	31	10.3
Feelings of pins and needles in arms or legs	19	6.3	45	14.9
Throat pain	17	5.6	67	22.1
Impaired vision	15	5.0	30	9.9
Problems with coordination of movements	13	4.3	27	8.9
Pink eyes or conjunctivitis	12	4.0	15	5.0
Nausea or vomiting	10	3.3	13	4.4
Swallowing pain	9	3.0	41	13.6
Loss of appetite	8	2.6	19	6.3
Shivering	5	1.7	12	4.0
Increased temperature	5	1.7	18	5.9
Blue lips	4	1.3	4	1.3
Fever (38.1 °C or higher)	2	0.7	12	4.0
Haemoptysis	0	0	0	0
